# Factors influencing parental decisions to terminate pregnancies following prenatal diagnoses of major fetal anomalies at Ramathibodi Hospital, Bangkok, Thailand

**DOI:** 10.1186/s12884-022-04813-w

**Published:** 2022-06-13

**Authors:** Papapin Pusayapaibul, Jittima Manonai, Chayada Tangshewinsirikul

**Affiliations:** 1 Department of Obstetrics and Gynecology, Faculty of Medicine, Ramathibodi Hospital, Mahidol University, Bangkok, Thailand; 2 Division of Maternal-Fetal Medicine, Department of Obstetrics and Gynecology, Faculty of Medicine, Ramathibodi Hospital, Mahidol University, Bangkok, 10400 Thailand

**Keywords:** Parental decision making, Fetal anomalies, Termination of pregnancy

## Abstract

**Background:**

The rate of termination of pregnancy (TOP) for fetal anomalies and the factors affecting TOP vary among different populations. Optimisation of prenatal care and counselling requires understanding the factors influencing parental decisions in the relevant population. This study aimed to evaluate the rate of TOP after diagnoses of major fetal anomalies and assess factors associated with TOP-related decisions at a university hospital in Thailand.

**Methods:**

A retrospective chart review was conducted at the Fetal Anomaly Clinic of Ramathibodi Hospital, Bangkok, Thailand. Medical records of all women with singleton pregnancies prenatally diagnosed with major fetal anomalies before 24 gestational weeks between 2010 and 2020 were reviewed.

**Results:**

During the study period, 461 cases of major fetal anomalies were diagnosed, and 264 (57.3%) of these pregnancies were terminated. Three factors influencing parental TOP decisions were lethal anomalies (odds ratio [OR], 197.39; 95% confidence interval [CI], 49.95–779.95; *p* < 0.001), presence of genetic abnormalities (OR, 10.19; 95% CI, 4.17–24.87; *p* < 0.001) and gestational age at diagnosis (OR, 0.74; 95% CI, 0.65–0.84; *p* < 0.001).

**Conclusions:**

Over half of the pregnant women whose records were reviewed and who were prenatally diagnosed with major fetal anomalies terminated their pregnancies. Fetal factors, particularly lethality, genetic abnormalities and early gestational age at diagnosis, showed the most powerful associations with parental TOP decisions. Other maternal background factors were not key considerations.

## Background

Advanced knowledge, prenatal ultrasounds and prenatal diagnoses have significantly improved the detection of fetal anomalies, allowing obstetricians to provide antenatal management plans, including fetal surveillance, fetal therapy and maternal transfer for delivery at tertiary centres offering effective postnatal treatment [1]. Congenital anomalies are major aetiologies of neonatal death worldwide. Some surviving infants risk having disabilities and/or developmental delays that impose burdens on themselves and their parents. These outcomes are particularly concerning in developing countries because of the low availability of and accessibility to healthcare systems and because facilities allowing these patients to have a suitable quality of life are insufficient. Consequently, termination of pregnancy (TOP) in cases of severe anomalies becomes an option for parents.

In Thailand, the prevalence of major congenital anomalies is 26.12 per 1000 live births and accounts for approximately one-fifth of neonatal deaths [2]. Thailand is a middle-income developing country, and most of its population is Buddhist. The national health policy provides maternal serum screening and mid-trimester ultrasound screening for all patients and a prenatal diagnosis in indicated pregnant women. Thai Penal Code 305 and Thai Medical Council Regulation 2005 permit legal abortions for pregnant women experiencing severe stress from the risk of having a fetus with severe anomalies and/or genetic disorders as determined by a qualified physician [3]. Safe abortions are promoted for these women and are covered by national health insurance.

Several complicated factors, including parents’ psychological, sociocultural and religious backgrounds and economic status, affect parental decisions to continue or terminate a pregnancy. Previous studies have indicated that the TOP rate for fetal anomalies varies from 25 to 90% among populations with a variety of legal gestational limits [4–10]. Factors identified to affect parental decisions include maternal age, previous uncompleted pregnancies, rural residency, religious background, gestational age at diagnosis, severity of the fetal anomalies, involvement of the central nervous system or multiple organs and presence of genetic abnormalities [4–10].

Several reports have been published on parental decisions regarding TOP for fetal anomalies in developed countries; however, to our knowledge, similar studies with adequate case numbers and designs are scarce for developing countries, especially in the South-East Asia region, including Thailand [4–12]. This study was conducted to investigate the rates of TOP following diagnoses of major fetal anomalies before 24 gestational weeks at Ramathibodi Hospital, Bangkok, Thailand. A secondary goal was to evaluate the factors related to these parental decisions. This information may improve prenatal care and counselling in Thailand and South-East Asia.

## Methods

### Setting

The Fetal Anomaly Clinic (FAC) at Ramathibodi Hospital is a maternal-fetal medicine (MFM) clinic in a university hospital located in Thailand’s capital city. This institute employed 6–11 MFM specialists between 2010 and 2020, and five were present throughout the entire study period. Pregnant women with suspected fetal anomalies based on ultrasound with or without abnormal maternal serum screening or cell-free DNA screening results were referred for further management. Couples at risk for thalassemia and pregnant women desiring a prenatal diagnosis owing to advanced maternal age but without structural fetal anomalies are not referred to the FAC.

MFM fellows and staff performed specialised ultrasound scans for all patients using a Voluson E8 or E10 (GE Healthcare, Wauwatosa, WI, USA). Invasive diagnostic procedures, such as karyotyping, chromosomal microarray analysis, gene mutation tests and alpha thalassemia major testing, were offered for indicated patients and performed by MFM fellows and staff. If an anomaly was identified, the MFM team provided counselling regarding the nature of the disease, prognosis of the fetus and potential management options; termination of pregnancy or continuing pregnancy with close surveillance or fetal therapy as indicated, for the women and their husbands and/or relatives. In cases when the MFM staff lacked the confidence to advise about particular anomalies or parental requests, neonatologists, paediatric geneticists, paediatric cardiologists and paediatric surgeons were involved in the counselling. The severity grade of a fetal anomaly was documented by consensus between at least two MFM staff members.

Medical TOP was offered in severe cases. Parental decisions to continue or terminate the pregnancy were based on their autonomous and informed consent after counselling. TOP was not offered in less severe cases; however, when parental concerns were voiced, a committee of other physicians and MFM staff reached agreements on case-by-case bases.

### Materials and methods

This retrospective study included pregnant women diagnosed with major fetal anomalies at the FAC at Ramathibodi Hospital between January 2010 and February 2020. Women with singleton pregnancies who were diagnosed with major fetal anomalies prior to 24 gestational weeks (the limit for TOP at our institute since gestational age beyond this is considered as viable state in most tertiary centers in Thailand) were enrolled. Information about maternal and fetal variables, along with parental decisions, was extracted from the clinic and hospital medical records. Cases were excluded if they had incomplete data regarding ultrasound findings and/or parental decisions or if the pregnancies ended in miscarriage or fetal demise before a decision was made.

Comprehensive maternal characteristics included maternal age at diagnosis, religious background, education level, gravidity and receipt (or not) of counselling from a multidisciplinary team including both MFM specialists and at least one neonatologist, paediatric geneticist, paediatric cardiologist or paediatric surgeon. Fetal variables consisted of gestational age at diagnosis, presence of genetic abnormalities (chromosomal abnormalities and syndromes), affected organ system and severity of anomalies. For this study, fetal anomaly severities were classified into three groups based on the probability of perinatal death: ‘lethal’ (> 50%), ‘potentially lethal’ (15–50%) and ‘non-lethal’ (< 15%) [6, 13]. The primary outcome in this study was the TOP rate; secondary outcomes were factors associated with the parental decisions for TOP. The Ramathibodi Hospital Institutional Review Board (COA. MURA2020/504) approved the study, which complied with the Declaration of Helsinki.

#### Statistical methods

Statistical analyses were performed using STATA, version 17 (STATA Corp, College Station, TX, USA). Parametric continuous variables are expressed as the mean ± standard deviation and were compared using Student’s t-test. Non-parametric continuous data are expressed as the median and interquartile range and were compared using the Mann-Whitney U test. Categorical variables were defined as the number (percent) and compared using the chi-square or Fisher’s exact test. *P <* 0.05 was considered statistically significant. Associations between maternal and fetal variables and parental decisions were tested using univariate and multivariate regression analyses.

## Results

**Fig. 1:**
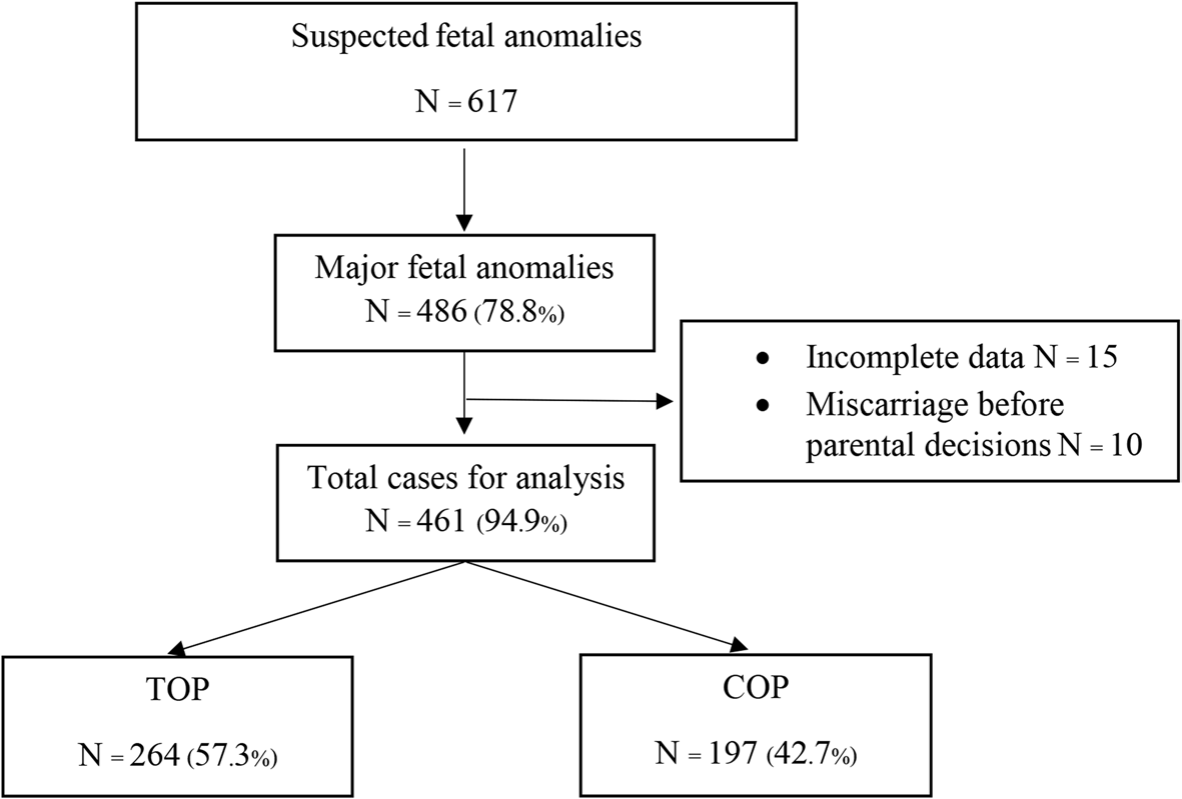
Total cases of major fetal anomalies and parental decisions. COP, continuation of pregnancy. TOP, termination of pregnancy

During the study period, 617 cases were referred to the FAC for suspected fetal anomalies at a gestational age of < 24 weeks. Of those, 486 cases (78.8%) involving major fetal anomalies were enrolled. Fifteen cases with incomplete data and ten that ended in miscarriage before parental decisions were excluded, leaving 461 cases (94.9%) for the final analysis. Parents chose TOP in 264 cases (57.3%) and continued the pregnancy in 197 cases (42.7%) (Fig. 1).

Among the 461 cases, the mean maternal age at diagnosis was 31.6 ± 6.1 years. Most patients were Buddhist. The median gestational age at diagnosis was 20 weeks (IQR 11–23 weeks). The three most common organ abnormalities were cardiovascular (31.9%), central nervous system (15.2%) and genitourinary (10.6%). Half the anomalies (51.4%) were classified as lethal. Genetic abnormalities accounted for 132 cases (28.6%); of these, 124 had chromosomal abnormalities (93.9%), one had Alagille syndrome, two had homozygous haemoglobin Constant Spring and five had Bart’s hydrops fetalis (Table 1).

**Table 1:** Demographics and fetal characteristics of pregnant women diagnosed with major fetal anomalies at a Fetal Anomaly Clinic

Characteristic	N	%
Maternal age (years)		
< 35	302	65.5
≥ 35	159	34.5
Religion		
Buddhist	420	92.7
Muslim	23	5.1
Christian	10	2.2
Educational level		
High school or less	181	40.3
Bachelor’s degree	221	49.2
Higher than a bachelor’s degree	47	10.5
Geographic origin		
Central and capital city	186	43.3
Northern	26	6.1
Southern	46	10.7
North-eastern	133	30.9
Other	39	9.1
Gravidity		
Primigravida	184	39.9
Multigravida	277	60.1
Method of conception		
Natural conception	455	98.7
ART	6	1.3
Had at least one child		
Yes	211	45.8
No	250	54.2
Previous history of abortion		
Yes	121	26.3
Induced abortion	30	24.8
Therapeutic abortion	91	75.2
No	340	73.8
Previous history of having a child with an anomaly		
Yes	25	5.4
No	436	94.6
Organ system		
Isolated	310	67.3
CNS	47	15.2
Face	24	7.7
CVS	99	31.9
Respiratory system	18	5.8
GI	10	3.2
Abdominal wall	16	5.2
GU	33	10.6
Skeletal	22	7.1
Soft tissue	30	9.7
Others	11	3.6
Multiple	151	32.8
Severity grade		
Lethal	237	51.4
Potentially lethal	149	32.3
Not lethal	75	16.3
Genetic abnormalities		
Yes	132	28.6
No	174	37.7
Unknown	155	33.6
Counselling by multidisciplinary team		
Yes	80	17.4
No	381	82.6

*ART* assisted reproductive technology, *CNS* central nervous system, *CVS* cardiovascular system, *GI* gastrointestinal, *GU* genitourinary

Table 2 shows the fetal anomaly groupings by severity grade and parental decisions to terminate the pregnancies. The fetal anomalies with TOP rates of 100% were anencephaly, bilateral renal agenesis or severe urinary tract obstruction, limb body wall complex, meningocele or encephalocele, trisomy 13, monosomy X with hydrops fetalis and Bart’s hydrops fetalis.

**Table 2:** Fetal anomaly grouping by severity grade and parental decisions to terminate pregnancy

Fetal anomalies	Total (*n* = 461) N	TOP (*n* = 264) N (%)
Lethal	237	201 (84.8)
Anencephaly	16	16 (100)
Hypoplastic left heart syndrome	8	7 (87.5)
Multiple cardiac anomalies	25	14 (56)
Bilateral renal agenesis or severe urinary tract obstruction	6	6 (100)
Lethal skeletal dysplasia	9	7 (77.8)
Limb body wall complex	7	7 (100)
Bart’s hydrops fetalis	5	5 (100)
Multiple organ defects	72	55 (76.4)
Chromosomal abnormalities	79	77 (97.5)
- Trisomy 13	11	11 (100)
- Trisomy 18	38	36 (94.7)
- Monosomy X with hydrops fetalis	12	12 (100)
- Other chromosomal abnormalities with multiple anomalies	18	18 (100)
Others	10	7 (70)
Potentially lethal	149	59 (39.6)
Meningocele or encephalocele	4	4 (100)
Hydrocephalus	8	4 (50)
Other CNS anomalies	5	3 (60)
Cardiac defects	36	6 (16.7)
Congenital diaphragmatic hernia	7	3 (42.9)
Congenital pulmonary airway malformation	10	0
Abdominal wall defects	16	0
Urinary tract obstruction with normal amniotic fluid	4	0
Non-lethal skeletal dysplasia	3	0
Chromosomal abnormalities	42	36 (85.7)
- Trisomy 21 with major structural anomalies	31	29 (93.6)
- Other chromosomal abnormalities	11	7 (63.6)
Others	14	3 (21.4)
Non-lethal	75	4 (5.3)
Cleft lip and/or palate	24	0
Cystic hygroma	8	1 (12.5)
Club foot	3	0
Ambiguous genitalia	3	0
Trisomy 21 alone with minor anomalies	3	3 (100)
Others	34	0

Table 3 shows the associations between maternal and fetal characteristics and parental decisions. The TOP rate was significantly associated with seven factors: maternal age (*p* < 0.001), gravidity (*p* < 0.001), gestational age at diagnosis (*p* < 0.001), multiple anomalies (*p* < 0.001), central nervous system anomalies (*p* < 0.001), severity of major anomalies (*p* < 0.001) and presence of genetic abnormalities (*p* < 0.001). No other characteristics significantly influenced the parental assessments (*p* > 0.05).

**Table 3:** Associations between maternal and fetal characteristics and parental decisions

Characteristics	TOP N (%)	COP N (%)	*p*-value	OR (95%CI)
Maternal age (mean ± SD)	32.8 ± 5.9	29.9 ± 6.1	< 0.001	
Maternal age (years)			< 0.001	2.25 (1.5–3.38)
< 35	153 (58)	149 (75.6)		
≥35	111 (42.1)	48 (24.4)		
Education			0.103	1.37 (0.94–2.01)
< Bachelor’s degree	94 (37)	87 (44.6)		
≥ Bachelor’s degree	160 (63)	108 (55.4)		
Gravidity			0.029	1.52 (1.04–2.22)
Primigravida	94 (35.6)	90 (45.7)		
Multigravida	170 (64.4)	107 (54.3)		
Method of conception			0.717	1.35 (0.27–6.74)
Natural conception	261 (98.9)	194 (98.5)		
ART	3 (1.1)	3 (1.5)		
Had at least one child			0.431	1.16 (0.8–1.68)
Yes	125 (47.4)	86 (43.7)		
No	139 (52.7)	111 (56.4)		
Previous history of abortion			0.314	1.24 (0.81–1.9)
Yes	74 (28)	47 (23.9)		
No	190 (72)	150 (76.1)		
Previous history of having a child with an anomaly			0.584	1.25 (0.56–2.81)
Yes	13 (4.9)	12 (6.1)		
No	251 (95.1)	185 (93.9)		
GA at diagnosis (weeks), median (IQR)	20 (11–23)	21 (12–23)	< 0.001	0.77 (0.71–0.84)
Organ system			< 0.001	9.37 (5.51–15.93)
Isolated	132 (50)	178 (90.4)		
Multiple	132 (50)	19 (9.6)		
CNS anomalies			< 0.001	5.69 (2.77–11.70)
Yes	36 (27.3)	11 (6.2)		
No	96 (72.7)	167 (93.8)		
Severity grade			< 0.001	
Lethal	201 (76.1)	36 (18.3)		99.10 (34.07–288.30)
Potentially lethal	59 (22.4)	90 (45.7)		11.64 (4.03–33.56)
Non-lethal	4 (1.5)	71 (36)		
Genetic abnormalities			< 0.001	
Yes	121 (45.8)	11 (5.6)		8.37 (4.18–16.77)
No	55 (20.8)	119 (60.4)		0.35 (0.22–0.55)
Unknown	88 (33.3)	67 (34)		

*ART* assisted reproductive technology, *CI* confidence interval, *CNS* central nervous system, *COP* continuation of pregnancy, *GA* gestational age, *IQR* interquartile range, *OD* odds ratio, *SD* standard deviation, *TOP* termination of pregnancy

Table 4 shows the multivariate logistic regression analysis of the maternal and fetal variables that affected parental decisions for TOP. Classification of fetal abnormalities as lethal (odds ratio [OR], 197.39; 95% confidence interval [CI], 49.95–779.95; *p* < 0.001) or potentially lethal (OR, 16.35; 95% CI, 4.35–61.43; *p* < 0.001), presence of genetic abnormalities (OR, 10.19; 95% CI, 4.17–24.87; *p* < 0.001) and gestational age at diagnosis (OR, 0.74; 95% CI, 0.65–0.84; *p* < 0.001) significantly independently affected parental decisions regarding TOP.

**Table 4:** Multivariate logistic regression analysis of association between maternal and fetal characteristics and parental decisions regarding termination of pregnancy

Characteristics	Multivariate analysis	
	Adjusted OR (95% CI)	*p*-value
GA at diagnosis	0.74 (0.65–0.84)	< 0.001
Severity grade		
Lethal	197.39 (49.95–779.95)	< 0.001
Potentially lethal	16.35 (4.35–61.43)	< 0.001
Genetic involvement		
Abnormal	10.19 (4.17–24.87)	< 0.001
Normal	0.32 (0.17–0.59)	0.001

*CI* confidence interval, *GA* gestational age, *OR* odds ratio

## Discussion

When major fetal anomalies are diagnosed, optimisation of prenatal counselling for pregnant women requires understanding the factors affecting parental decisions to terminate or continue the pregnancy in the relevant population. In this study, the rate of TOP owing to fetal anomalies was 57.3%, which is higher than that reported in most previous studies with similar legal gestational age limits [4–6]. Data from the New Jersey Fetal Abnormalities Registry, which has a majority white population, showed a 33% rate of TOP when fetal defects were identified before 24 gestational weeks [5]. One explanation for the higher TOP rate in our study may have been that the rate of lethal fetal anomalies, a significant factor affecting parents’ decisions, was greater than that in the New Jersey study (51.4% vs 27.8%).

The lack of an efficient congenital anomalies registration system in developing countries, especially in South-East Asia, leads to inadequate information regarding TOP rates for fetal anomalies, prenatal diagnosis rates, and fetal and neonatal outcomes. Interestingly, Ho et al. reported no incidence of TOP for 38 fetal anomalies detected during the antenatal period in Malaysia and concluded that TOP for fetal anomalies was infrequent in developing countries [12]. However, the important limitations of that study were its small number of cases and a failure to mention the gestational age at prenatal diagnosis and the legal gestational limit in Malaysia. In our study, over half of the 461 prenatal patients elected TOP when the fetal anomalies were detected before 24 gestational weeks. These different results from countries in the same region illustrate the necessity of an effective data collection and registration system for congenital anomalies in developing countries. This information could help improve the quality of prenatal care systems in this region.

Expectedly, potentially lethality or lethality and genetic abnormalities played important roles in the parental TOP decisions, and we found a strong predictive relationship between these two factors and TOP [5, 6, 9, 11]. Various techniques for fetal therapy have been developed but are not generally accessible, and treatment outcomes are often unsatisfactory, especially in developing countries. Furthermore, in low-resource countries, health care systems for children with congenital anomalies are often inaccessible because specialists are limited to a few university hospitals. Moreover, patients with disabilities in developing countries often have a poor quality of life [14]. Consequently, parental consideration of the quality of life for their children with severe birth defects, the potential for disability and/or developmental delays, and the burden of caring for these children may guide parents to elect TOP [15].

Considering the influence of fetal lethality on parental decisions, lethal and potentially lethal anomalies, anencephaly, bilateral renal agenesis or severe urinary tract obstruction, meningocele or encephalocele, monosomy X with hydrops fetalis and Bart’s hydrops fetalis had TOP rates of 100% in our study (Table 2). Surprisingly, a study from Israel found that the TOP rates for these same anomalies diagnosed before 24 gestational weeks were only 22–50% [4]. The only profound predictors of termination decisions in that study were an earlier gestational week at diagnosis and previous uncompleted pregnancies; presumed fetal lethality was not a predictor. This difference demonstrates that TOP rates and factors affecting parental decisions vary among countries depending on the TOP laws and on the population backgrounds.

Early diagnosis was an important independent factor in the parents’ decisions. Patients with an earlier diagnosis of fetal anomalies tended to terminate the pregnancy more frequently than did those with a later diagnosis. Our findings are consistent with previous studies from developed countries [4, 5, 7], possibly because the maternal-fetal bonding that occurs as the gestational weeks progress makes the TOP decision more emotional and difficult for parents. Later diagnoses also make the TOP more difficult and increase the risk of complications [1, 16]. In Thailand, ultrasound anatomy screening at 18–22 gestational weeks is a national policy, and some fetal anomalies can be detected as early as the first trimester [17]. Therefore, pregnant women should be encouraged to obtain early antenatal care. Additionally, obstetricians should be broadly educated and trained in early comprehensive ultrasounds and prenatal diagnoses to detect high-risk cases because earlier diagnosis of fetal anomalies, as well as genetic abnormality involvement, affect parents’ management plans.

A semi-structured interview study by Phaophan et al. showed that having a Buddhist background did not influence the decisions of pregnant Thai women to terminate their pregnancy when fetal ß-thalassemia was detected [18]. Nevertheless, since Buddhism has no strict guidelines on abortion issues, attitudes toward abortion appear vary between pregnant women. The primary religious background of the pregnant women in our study was Buddhist, and all data were declared and collected from patients’ medical records; however, these data may not represent patients’ actual beliefs. Consequently, to fully understand the patients in this region, further qualitative studies with in-depth interviews should be conducted to determine the factors, especially religious and sociocultural factors, associated with parental decisions.

Because of the scarcity of other subspecialists and the complexity of the interdepartmental referral system within the pregnancy timeframe, only 17% of our couples received counselling by a multidisciplinary team. Although discussions with specialists who have their own expertise and different perspectives can improve parental decision making, especially regarding complex or uncommon fetal anomalies, in terms of ethical and management options during the prenatal and postnatal periods, the New Jersey study stated that genetic counselling did not appear to play an important role in parental decisions [5, 19–21]. This may reflect the high-quality targeted counselling and the substantial amount of time with MFM specialists available in the U.S. Therefore, the counselling techniques of the MFM team should be improved, and interdepartmental referral systems for fetal anomaly cases should be coordinated. The effect of a multidisciplinary team approach on parental decisions and satisfaction in developing countries requires further studies.

This study is the first to report the TOP rate and the factors that influence it for fetal anomalies in Thailand, a middle-income developing country. The results highlighted that fetal information, including gestational age, structural abnormalities and genetic involvement, should be evaluated and considered during prenatal care and counselling. Therefore, a comprehensive fetal anatomical scan and prenatal diagnosis at as early as 18–22 gestational weeks should be offered in antenatal care clinics.

The main limitation of this study was its retrospective nature, which precluded any control over the counselling techniques used by the various MFM staff over the past 10 years. A non-directive method is the standard technique; however, each counsellor may have their own convictions and perspectives regarding TOP, as well as varying counselling skills, which could affect parental decisions [21]. Another limitation was that the results were from a single university hospital in the capital city of Thailand; hence, these results may not be generalisable to hospitals in other parts of Thailand or to other countries with different sociocultural backgrounds. However, pregnant women from regions nationwide travelled to the capital city and enrolled in this study; therefore, the findings may represent those of the entire Thai population and could correspond to other developing countries to some extent.

## Conclusions

Over half of the pregnant women enrolled in this study who were prenatally diagnosed with major fetal anomalies terminated their pregnancies. This was particularly the case for fetuses with lethal anomalies, genetic abnormalities and early gestational ages. Comprehensive fetal ultrasounds and prenatal diagnoses as early as 18–22 gestational weeks should be offered in antenatal care clinics.

## Data Availability

The datasets generated and/or analysed during the current study are not publicly available due to limitations of ethical approval involving the patient data and anonymity but are available from the corresponding author on reasonable request.

## Abbreviations

TOP: Termination of pregnancy
FAC: Fetal Anomaly Clinic
MFM: Maternal-fetal medicine
OR: Odds ratio
CI: Confidence interval
